# Exercise-Associated Pathways as Novel Neuroprotectants Against CNS Aging and Alzheimer’s Disease

**DOI:** 10.1093/geroni/igab046.1440

**Published:** 2021-12-17

**Authors:** Constanza Cortes

**Affiliations:** University of Alabama at Birmingham, University of Alabama at Birmingham, Alabama, United States

## Abstract

Skeletal muscle has recently arisen as a novel regulators of Central Nervous System (CNS) function and aging, secreting bioactive molecules known as myokines with proteostasis and metabolism-modifying functions in targeted tissues. We have recently generated a novel transgenic mouse with enhanced muscle proteostasis via moderate overexpression of Transcription Factor E-B (TFEB), a powerful master regulator of cellular clearance and proteostasis. We have discovered that the resulting enhanced skeletal muscle proteostasis function can significantly ameliorate proteotoxicity in the aging CNS and improve cognition and memory in aging mice. These neuroprotective benefits are markedly reminiscent of those observed in the aging CNS post-exercise, suggesting enhancing muscle proteostasis may be sufficient to replicate the local and systemic effects of exercise. Identification of pathways regulating crosstalk between skeletal muscle and CNS may yield targets with high therapeutic potential for diseases of the aging CNS.

